# Enhancement of Peripheral Nerve Regrowth by the Purine Nucleoside Analog and Cell Cycle Inhibitor, Roscovitine

**DOI:** 10.3389/fncel.2016.00238

**Published:** 2016-10-17

**Authors:** Vincent Law, Sophie Dong, Jesusa L. Rosales, Myung-Yung Jeong, Douglas Zochodne, Ki-Young Lee

**Affiliations:** ^1^Department of Cell Biology and Anatomy, Arnie Charbonneau Cancer Institute, Hotchkiss Brain Institute, University of CalgaryCalgary, AB, Canada; ^2^Department of Clinical Neurosciences, Hotchkiss Brain Institute, University of CalgaryCalgary, AB, Canada; ^3^Department of Biochemistry and Molecular Biology, Snyder Institute for Chronic Diseases, University of CalgaryCalgary, AB, Canada; ^4^Department of Cogno-Mechatronics Engineering, Pusan National UniversityPusan, South Korea

**Keywords:** peripheral nerve, injury, axon, regeneration, cytoskeleton

## Abstract

Peripheral nerve regeneration is a slow process that can be associated with limited outcomes and thus a search for novel and effective therapy for peripheral nerve injury and disease is crucial. Here, we found that roscovitine, a synthetic purine nucleoside analog, enhances neurite outgrowth in neuronal-like PC12 cells. Furthermore, *ex vivo* analysis of pre-injured adult rat dorsal root ganglion (DRG) neurons showed that roscovitine enhances neurite regrowth in these cells. Likewise, *in vivo* transected sciatic nerves in rats locally perfused with roscovitine had augmented repopulation of new myelinated axons beyond the transection zone. By mass spectrometry, we found that roscovitine interacts with tubulin and actin. It interacts directly with tubulin and causes a dose-dependent induction of tubulin polymerization as well as enhances Guanosine-5′-triphosphate (GTP)-dependent tubulin polymerization. Conversely, roscovitine interacts indirectly with actin and counteracts the inhibitory effect of cyclin-dependent kinases 5 (Cdk5) on Actin-Related Proteins 2/3 (Arp2/3)-dependent actin polymerization, and thus, causes actin polymerization. Moreover, in the presence of neurotrophic factors such as nerve growth factor (NGF), roscovitine-enhanced neurite outgrowth is mediated by increased activation of the extracellular signal-regulated kinases 1/2 (ERK1/2) and p38 mitogen-activated protein kinase (MAPK) pathways. Since microtubule and F-actin dynamics are critical for axonal regrowth, the ability of roscovitine to activate the ERK1/2 and p38 MAPK pathways and support polymerization of tubulin and actin indicate a major role for this purine nucleoside analog in the promotion of axonal regeneration. Together, our findings demonstrate a therapeutic potential for the purine nucleoside analog, roscovitine, in peripheral nerve injury.

## Introduction

Unlike in the central nervous system, some degree of axonal regeneration and functional recovery is possible after injury in the peripheral nervous system. However, the process is slow and many times incomplete. Following injury, Wallerian-like degeneration (WLD) occurs in the denervated distal stump where myelin and myelin-associated glycoproteins are broken down to allow axonal regeneration (Stoll et al., 2002). This event correlates with upregulation of cytosolic and secreted forms of phospholipase A2 in Schwann cells (SC; De et al., 2003), which undergo dedifferentiation to promote a change in the damaged nerve environment to support neuron survival and axon regrowth (Jessen and Mirsky, 2008).

Axonal regrowth is one of the cellular processes that require modulation of actin and tubulin polymerization (Eng et al., 1999; Richardson et al., 2009). Indeed, during injured peripheral nerve recovery, the growth cone depends on the upregulation of tubulin subunits in its core and in the core of the trailing axon as well as actin, which forms the filopodia. Thus, tubulin and actin form the major cytoskeletal framework of elongating axons of the regenerating nerve (Chierzi et al., 2005; Vogelaar et al., 2009).

A multitude of endogenous and exogenous factors have been demonstrated to have an impact on the speed and direction of growth of regenerating axons. For example, the naturally occurring purine nucleoside, inosine, has been implicated in collateral axon growth in the corticospinal tract after focal traumatic brain injury and stroke (Dachir et al., 2014). In addition, the purine nucleosides, adenosine and guanosine, have been suggested to influence axonal degeneration after injury of dorsal root ganglia neurons (Press and Milbrandt, 2009). Furthermore, adenosine was found to aid axon preservation after injury (Press and Milbrandt, 2009). These observations suggest that purine nucleosides may have the ability to protect axon integrity.

A synthetic purine analog, R-roscovitine (also known as CYC202 and Seliciclib), interacts with a number of proteins, including cyclin-dependent kinases (Cdks), camodulin-dependent protein kinases (CaM kinases) and extracellular signal-regulated kinases (ERK; Bach et al., 2005), and has a varying inhibitory effect on these kinases (Meijer et al., 1997; Planchais et al., 1997). Roscovitine is particularly known as an inhibitor of Cdks, which are key regulators of cell cycle progression. Interestingly, inhibiting the cell cycle has been shown to cause decreased neuronal death as well as improved sensory motor recovery following spinal cord injury (Wu et al., 2011), and purine nucleosides have been demonstrated to protect axon integrity (Press and Milbrandt, 2009) and support axon growth (Dachir et al., 2014). However, there have also been studies showing that roscovitine inhibits neurite outgrowth (Harada et al., 2001; Lee and Kim, 2004; Carter et al., 2008). Thus, the role of roscovitine in neurite outgrowth remains to be elucidated.

Although roscovitine inhibits mitogen-activated protein kinase (MAPKs), it is a poor inhibitor of these kinases with IC_50_ values of 34 μM, 14 μM and >10 μM for ERK1, ERK2 and p38 MAPK, respectively, in *in vitro* purified enzyme assays (Meijer et al., 1997). The molecular mechanism by which roscovitine inhibits protein kinase activity involves competition for adenosine 5′-triphosphate (ATP)-binding pockets within the enzyme (De Azevedo et al., 1997; Gray et al., 1999). The binding of roscovitine is a reversible process, and based on pharmacokinetic studies using intravenous injections in mouse and rat models, it shows a high degree of dissemination and rapid metabolomic degradation (Vita et al., 2004; Nutley et al., 2005; Raynaud et al., 2005). Interestingly, roscovitine is currently being explored as a therapeutic agent for specific types of cancer such as non-small cell lung cancer (Hamilton et al., 2014) and nasopharyngeal cancer (Hui et al., 2009) as well as therapy for Cushing’s disease (Liu et al., 2015).

Previously, roscovitine has been shown to rescue actin stress fibers formation in Rat-2 fibroblast cells that overexpress CKIγ2. It was thought that this was achieved through a Cdk-independent p27^KIP^ pathway (Latreille et al., 2012). Roscovitine has also been correlated with microtubule formation in mitotic *S2* *Drosophila* cells. It was believed that this correlation involved inactivation of Cdk1 (Moutinho-Pereira et al., 2010). In yet another study, roscovitine was associated with the formation of cytoskeletal aggregates in apoptotic bodies in human CHP212 neuroblastoma cells (van Engeland et al., 1997). Based on these findings, we came to the hypothesis that roscovitine may interact with cytoskeletal components, particularly actin and tubulin, and that it is possible that following peripheral nerve injury, exposure to roscovitine could result in the alteration of actin and tubulin dynamics and subsequently promote axonal regeneration.

Previous investigations on the signaling cascades that regulate neurite outgrowth and nerve regeneration following injury have demonstrated the involvement of ERK1/2 and p38 MAPK. While for example, it was found that the ERK and p38 MAPK inhibitors, PD98059 and SB203580, respectively, inhibited artemisinin-induced neurite outgrowth (Sarina et al., 2013). In separate studies of rat crushed sciatic nerves, it was also determined that ERK activity increased on days 3 and 7 post injury while AKT and p38 MAPK showed increasing activities from 3 to 28 days post injury (Yamazaki et al., 2009). These findings are consistent with the idea that activation of ERK, p38 MAPK and AKT are involved in neurite outgrowth and axon regeneration.

In this study, we provide new insight into the targeting of both actin and tubulin by roscovitine. We demonstrate the ability of roscovitine to interact with actin and tubulin, and induce actin and tubulin polymerization. We also demonstrate that in addition to enhancing actin- and tubulin-dependent neurite outgrowth, roscovitine augments axonal regrowth and nerve regeneration following injury. Furthermore, we show that enhanced activation of ERK and p38 MAPK is linked to roscovitine-induced neurite outgrowth.

## Materials and Methods

### Animals and Preconditioning Lesion Experiments

Adult male Sprague-Dawley rats (Charles River Laboratory, Senneville, QC, Canada) with an initial weight of 300–500 g were used and all animal studies conformed to regulatory standards and were approved by the University of Calgary Health Sciences Animal Care Committee. For preconditioning, the sciatic nerve was cut at the mid-thigh region 3 days prior to harvesting dorsal root ganglia (DRG). A sham injury in separate animals was performed by exposing but not severing the sciatic nerve.

### PC12 Cell Culture, R-roscovitine Treatment and Neurite Outgrowth Analysis

PC12 cells were seeded at 1.5 × 10^5^ and 4.5 × 10^5^ (for western blot analysis) into 35 mm and 60 mm tissue culture dishes, respectively, in high serum-containing RPMI (10% horse serum, 5% fetal bovine serum) with penicillin-streptomycin. After 24 h, cells were subjected to serum starvation in RPMI containing 0.2% horse serum and penicillin-streptomycin for 18 h. Cells were then treated with 10 ng/ml nerve growth factor (NGF; Invitrogen Life Technologies), and/or graded concentrations (0.2, 2, 5, 10, 20 and 40 μM) of R-roscovitine (Invitrogen Life Technologies) for 24 h. Neurite outgrowth was examined by light microscopy (Olympus IX71) and images were analyzed using the Image-Pro Express software (Media Cybernetics). The length of neurites was analyzed using the Image-J software. Three optical fields and 50 cells/field were evaluated for each condition. Cell viability was assessed by trypan blue assay. Measurements were taken from 50 cells in each treatment (*n* = 3).

### Western Blot Analysis

For time-course assays, cells were exposed to 10 ng/ml NGF alone, 10 μM roscovitine alone, or both, and lysed at 0, 1, 3, 10, 30, 90 and 180 min post-treatment. Equal amounts of protein samples were then resolved in 12.5% sodium dodecyl sulfate- polyacrylamide gel electrophoresis (SDS-PAGE) and subjected to western blot analysis using total or phospho-antibodies against ERK1/2, p38 MAPK and AKT (Cell Signaling Technology, Inc.). Cdk5 and HRP-conjugated secondary antibodies were from Santa Cruz Biotechnology, Inc. and Invitrogen Life Technologies, respectively.

### Primary DRG Sensory Neuron Culture

Dissociated adult sensory neuron culture protocols using rat DRG were as described previously (Christie et al., 2014). Briefly, rats were anesthetized with Isoflurane (Abbot Laboratories) and then killed 3 days following the conditioning lesion or sham surgery. L4–L6 DRGs were removed from the rats and placed into L15 (Invitrogen) medium. The DRGs were rinsed and then transferred to a tube containing 2 ml 0.1% collagenase (Invitrogen)/L15. Following incubation at 37^o^C, the DRGs were placed into single-cell suspension by triturating. The single-cell suspension was spun and washed three times in 2 ml L15. After the final spin, the cells were resuspended in L15 and passed through a 70 mm mesh (VWR International Co.) and then placed in 500 ml L15 enriched with 1:100 dilution of N2 supplement (Invitrogen) and 0.1% BSA (Sigma) and placed into a culture medium of Dulbecco’s Modified Eagle Medium/F12 (DMEM/F12; Invitrogen) + 1:100 dilution N-2 supplement, 0.5–0.8% BSA and 0.2 ng/ml NGF (Cedarlane Labs) plus Penicillin 50 U/ml, Streptomycin 50 U/ml (Invitrogen) and plated on poly-L-lysine (Sigma-Aldrich) and 10 μg/ml mouse laminin (Invitrogen)-coated cover slips. Cells were grown for 24 h prior to roscovitine treatment (0.2, 2 or 10 μM) for 24 h.

### Immunocytochemistry of Primary DRG Sensory Neurons

Roscovitine-treated and untreated neurons were washed with 1× Phosphate-buffered saline (PBS) and fixed with 4% paraformaldehyde (Invitrogen Life Technologies), stained with β-tubulin III antibody (1:200 dilution; Sigma-Aldrich Co.) in 1× PBS containing 10% goat serum and 0.3% Triton X-100 and Cy3-conjugated anti-mouse secondary antibody (1:200 dilution; Sigma-Aldrich Co.) in 1× PBS containing 10% goat serum and 0.3% Triton X-100. Images were captured under a Zeiss fluorescence microscope. Neurite length was analyzed by the MetaXpress software and by an observer blinded to their condition (Molecular Devices, Sunnyvale, CA, USA). Between 40 and 60 neurons were analyzed per condition. Three to four rats per condition were routinely used. Graph Pad Prism was used for statistical analysis. Student’s *t*-tests were carried out with the statistical significance set at *p* ≤ 0.05.

### Mass Spectrometry

R-roscovitine bound proteins were eluted from agarose beads by SDS sample buffer and briefly subjected to SDS-PAGE. Coomassie stained protein bands were cut and analyzed by LC-MS/MS on a Micromass Q-ToF-2TM mass spectrometer coupled with a Waters CapLC capillary HPLC (SAMS Centre, University of Calgary). The peptide and protein false discovery rates were set at 1%. A total of 22 roscovitine-interacting proteins were identified, i.e., proteins identified and quantified from at least two unique peptides. Keratin contaminants were not counted. Significance values were determined by Perseus tool using significance B. Proteins with ratio greater than 1.9 and significance B lesser than 0.1 were considered significantly enriched. Eleven proteins listed in Figure 4B satisfied these criteria.

### R-roscovitine Agarose Pull-Down Assay and Analysis of R-roscovitine Binding Proteins

Lysates of PC12 cells treated with 10 ng/ml NGF or 10 ng/ml NGF + 10 μM roscovitine for 24 h were pre-cleared with agarose beads for 20 min and subsequently incubated with R-roscovitine agarose beads (provided by Dr. L. Meijer, Station Biologique de Roscoff, CNRS UPR, France) for 40 min. Beads were washed with 50 mM Tris, pH 7.4, containing 5 mM NaF, 250 mM NaCl, 5 mM EDTA, 5 mM EGTA, 0.1% Triton X-100 and protease inhibitor cocktail. R-roscovitine-binding proteins were eluted from beads with 2× SDS-PAGE sample buffer and resolved by SDS-PAGE. Separated proteins were stained with Sypro-Ruby Red (Invitrogen Life Technologies) and detected using the BioDoc IT^TM^ Image System. To identify R-roscovitine-binding proteins, pulled-down samples were subjected to SDS-PAGE and ran into the 12.5% gel. Protein bands were cut and analyzed by LC-MS/MS (SAMS Centre for Proteomics, University of Calgary).

### Nerve Regeneration Analysis

Axon regrowth was analyzed in transected rat sciatic nerve as described previously (Christie et al., 2010). An incision (3 cm) was made on the lateral region of the left thigh from the sciatic notch to the knee area. Another incision on the dorsal region superior to the scapulae and parallel to the vertebrae was performed to enable insertion of a microinjection port (MIP). Forceps were used to create a channel through the subdermal fascia between the incisions. Forceps were also used to pull the MIP catheter from the anterior site to the posterior incision. The MIP catheter was then attached to the T-chamber access tube. The left sciatic nerve was exposed and mobilized by blunt dissection, and transected at the mid-thigh region with a scalpel blade (11-0). The nerve stumps were attached to the nerve chamber with a nylon suture (Ethicon, 9-0) through the epineurium. A 3 mm gap was left between the proximal and distal stumps. Single sutures (Ethicon, 4-0) were used to attach the tube’s T-intersection to the underlying muscle and to reattach the retracted gluteal muscle. A continuous suture (4-0) was performed to close the incisions. On days 2, 4 and 6 following nerve injury, 100 μl of 10 μM roscovitine was administered. At the experimental end points, both the proximal and distal nerve segments were cut a few millimeters away from the nerve chamber. The epineurial sutures were removed to free the nerve bridge (regenerate), which was harvested by pulling it through the chamber. Four of the nine rats were sacrificed to detect early regenerating fibers 7 days post-injury. The rest (*n* = 5) of the animals were analyzed for regenerating myelinated axons 21 days post-injury. Immunohistochemistry was performed on bridges spanning the sciatic nerve transections using mouse NF200 (1:800; Sigma) and rabbit glial fibrillary acid protein (GFAP; 1:250; Dako) antibodies. Transected bridges were fixed with modified Zamboni’s solution overnight at 4°C then rinsed in PBS and suspended in PBS-20% sucrose solution overnight (4°C). Samples were then frozen in optimum cutting temperature (OCT; Tissue-Tek Sakura Finetek) and cryostat sections (12 μM) were placed on slides that were frozen at −80°C. For immunostaining, tissues were permeabilized with 0.3% Triton-X in 5% goat serum and 1% BSA for 1 h. Tissues were double labeled with NF-200 and GFAP, rinsed in PBS and incubated with secondary CY3 sheep anti-mouse (1:100, Sigma) and Alexa Fluor 488 goat anti-rabbit (1:500, Cedarlane) antibodies for 1 h. Tissues were then rinsed and mounted using Polyaquamount medium (Polysciences) and visualized using a Zeiss Axioscope with digital camera and Axiovision imaging software (Zeiss Axioskope, Axiovision, and Axiocam, Zeiss, North York, ON, Canada). Profiles of serial regions (every 270 mm) perpendicular to the direction of the bridge were examined and counted blindly.

### Epon Embedding and Sectioning

Segments of nerve are fixed in 2.5% glutaraldehyde in 0.025 M cacodylate buffer, washed with cacodylate buffer (0.15 M), postfixed (2% osmium tetroxide in 0.12 M cacodylate buffer), then dehydrated through graded ethanol and propylene oxide. Next, the nerve segments undergo infiltration with a 50/50 EPON/propylene oxide mixture overnight and are thereafter embedded in EPON and baked for 3 days (45°C for first day and then 65°C for final 2 days). Central segments of the nerve bridge (i.e., 1.5 mm from the proximal stump) are then cut into ~1 μM sections and stained with toluidine blue.

### Tubulin Polymerization Assay

Tubulin polymerization assay was performed using a kit, and with slight modification of the manufacturer’s (Cytokeleton Inc.) protocol. The recommendation for the *in vitro* tubulin polymerization assay kit is to use tubulin at 2 mg/ml in fluorescence buffer (80 mM PIPES pH 6.9, 2.0 mM MgCl_2_, 0.5 mM EGTA, 10 μM fluorescent reporter) + 1 mM Guanosine-5′-triphosphate (GTP) in black 96-well plates. As R-roscovitine is a synthetic GTP analog, a comparable amount of R-roscovitine was used: 0.2 mM or 0.4 mM when used alone or 0.4 mM when used together with 0.5 mM GTP. For control, Dimethyl sulfoxide (DMSO) and ddH_2_O were used instead of R-rsocovitine and GTP, respectively. The kinetics of tubulin polymerization was measured up to 60 min at 37°C using a spectrophotometer (SpectraMax M2, Molecular Devices; Softmax Pro software). Excitation and emission wavelengths were set at 355 nm and 460 nm, respectively.

### Purification of Strep-WAVE1

Strep-WAVE1 DNA construct (pAAV-CBA-ST-WAVE1) was transfected into COS7 cells using Lipofectamine 2000 (Invitrogen Life Technologies). After 48 h, transfected cells were lysed and Strep-WAVE1 was purified using the Strep-Tactic Spin Column (BioTAGnology) as described by Kim et al. (2006). Purification of WAVE1 was assessed by SDS-PAGE and Coomassie brilliant blue staining.

### Actin Polymerization Assay

Actin Polymerization assays were performed as described by the manufacturer (Cytokeleton Inc.). Pyrene-actin (40 μg) and non-muscle actin (20 μg) mixed in general actin buffer (0.2 mM ATP and 1 mM Dithiothreitol, DTT) were kept on ice for 1 h to allow depolymerization. For WAVE1 phosphorylation by Cdk5/p25, Strep-WAVE1 (36 nM), Actin-Related Proteins 2/3 (Arp2/3) protein complex (220 nM) were incubated with reconstituted Glutathione S-transferase (GST)-Cdk5/GST-p25 (16 μg) in 10 mM Tris-HCl buffer, pH 7.6, containing 50 μM ATP, 5 mM MgCl_2_, 50 mM KCl and 5 mM EGTA, for 30 min in a 30^o^C. To inhibit the activity of GST-Cdk5/p25, the strep-WAVE1 mixture was pre-incubated with 0.1 mM R-roscovitine before adding 50 μM ATP. An equal volume of DMSO was added to the control. For polymerization assays, pyrene-actin and non-muscle actin mix were aliquoted into black 96-well plates along with GST-Cdk5/GST-p25-phosphorylated WAVE1 (with or without 0.1 mM R-roscovitine) and Arp2/3 protein complex. Polymerization buffer (500 mM KCl, 20 mM MgCl_2_ and 10 mM ATP) was added to initiate polymerization. The kinetics of actin polymerization was measured up to 30 min using the SpectraMax M2 spectrophotometer at excitation and emission wavelengths of 355 nm was 405 nm, respectively.

## Results

### Roscovitine Enhances NGF-Mediated Neurite Outgrowth

**Figure 1:**
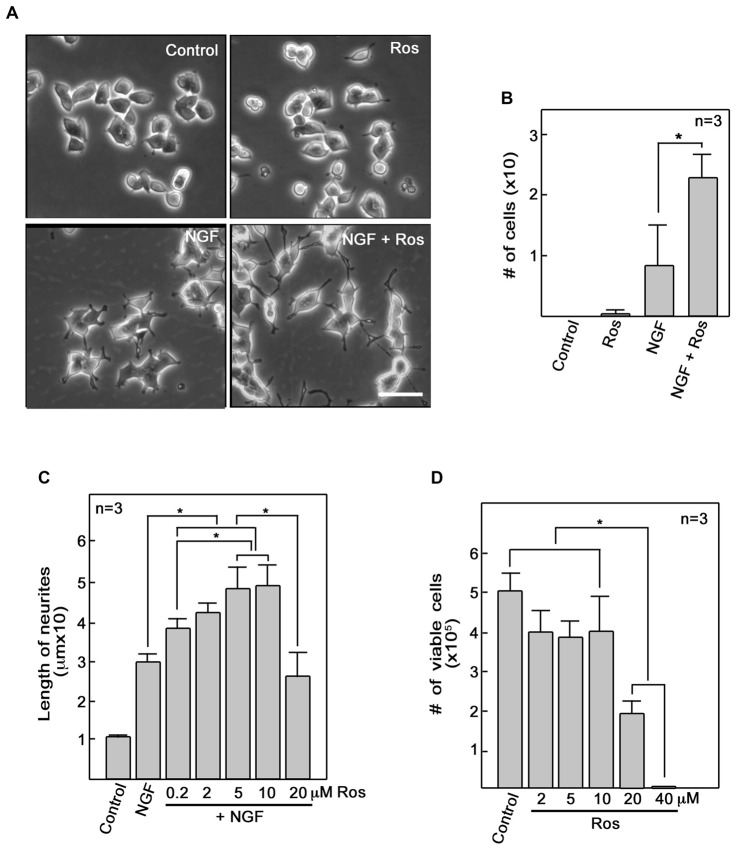
**Roscovitine enhances nerve growth factor (NGF)-mediated neurite outgrowth in PC12 cells.** **(A)** PC12 cells were plated as described in “Materials and Methods” Section. After 18 h in low serum DM, cells were treated with roscovitine alone (10 μM, upper right panel), NGF alone (10 ng/ml NGF; lower left panel), or NGF and roscovitine simultaneously (lower right panel). The upper left panel shows cells that were not treated with either roscovitine or NGF. Photographs were taken 24 h after treatment. Scale bar = 50 μM. **(B)** Shows the number of cells with neurites longer than 20 μM. Measurements were taken from 50 cells in each treatment (*n* = 3). Statistical significance using student’s *t*-test was set at *p* < 0.05*. **(C)** Neurite lengths of differentiating PC12 cells were measured 24 h after treatment with NGF in the absence or presence of roscovitine at various concentrations. Untreated cells were used as control. Values are means ± SD of three (*n* = 3) independent experiments. Statistical significance using student’s *t*-test was set at *p* < 0.05*. **(D)** Viability of cells treated with various concentrations of roscovitine was assessed after 24 h by cell counting using a hemocytometer. Values are means ± SD of three (*n* = 3) independent experiments. Statistical significance using student’s *t*-test was set at *p* < 0.05*.

To further investigate the role of roscovitine in neurite outgrowth, we initially used differentiating PC12 cells. We found that addition of roscovitine caused increased numbers of NGF-treated differentiating PC12 cells that exhibit longer neurites compared to those treated with NGF alone (Figures 1A,B). This is interesting but contradicts previous reports that roscovitine inhibits neurite outgrowth in PC12 cells (Harada et al., 2001; Lee and Kim, 2004; Carter et al., 2008). Since we used 10 μM roscovitine in our studies while others used 20–100 μM (Harada et al., 2001; Lee and Kim, 2004; Carter et al., 2008), we performed a roscovitine dose response analysis of NGF-treated differentiating PC12 cells. As shown in Figure 1C, it is clear that addition of roscovitine at 0.2–10 μM enhanced neurite lengths of NGF-treated cells (*p* < 0.05). Roscovitine at 0.2–10 μM caused a pattern of increasing neurite lengths, with 5 and 10 μM causing significantly (*p* < 0.05) greater length of neurites compared to that caused by 0.2 μM. However, a further increase in roscovitine concentration to 20 μM caused a drastic decrease (*p* < 0.05) in neurite length compared to treatment with 5 and 10 μM. Neurite length at 20 μM roscovitine is comparable to that of cells treated with NGF alone. While roscovitine has been shown to cause apoptosis (Gao et al., 2005; Zheng et al., 2007), we found that concentrations at which it can enhance neurite length (up to 10 μM) were insufficient to cause significant cell death (Figure 1D) but increasing the concentration to 20 and 40 μM caused loss of cell viability by ~60% and almost 100%, respectively (*p* < 0.05). Thus, our findings demonstrate that at lower concentrations, i.e., at 0.2–10 μM, roscovitine enhances neurite length instead of neuronal apoptosis.

### Roscovitine Enhances Axonal Regrowth

**Figure 2:**
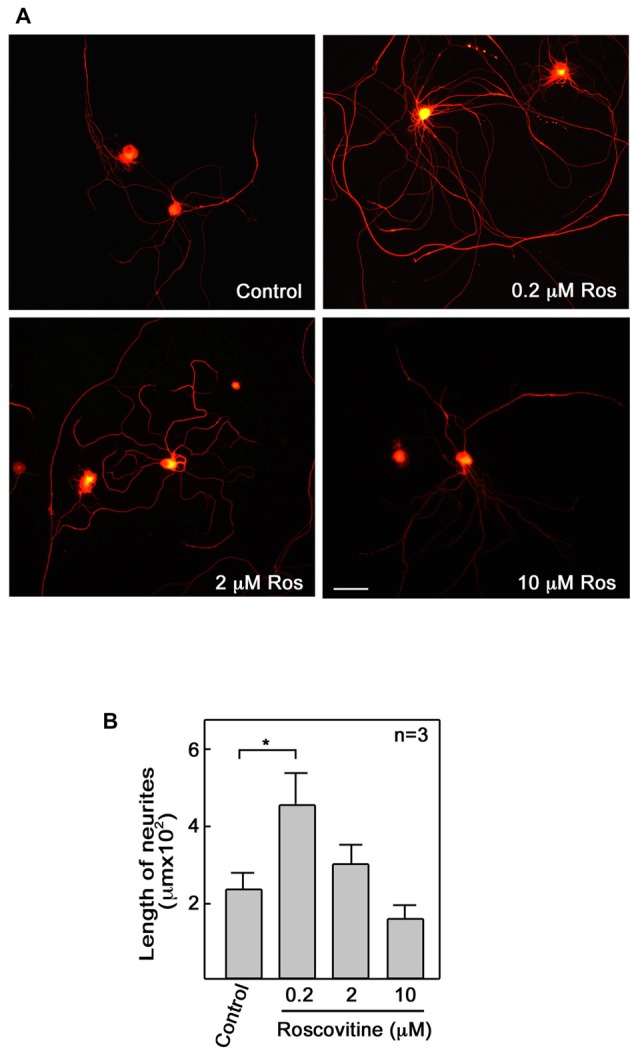
**Roscovitine enhances axonal regrowth of pre-injured rat primary dorsal root ganglia (DRG) sensory neurons. (A)** Neurons isolated from rat L4–L6 DRGs 3 days after sciatic nerve transection were cultured on laminin and poly-L-lysine coated cover slips in primary neuron media, containing N-2 supplement, BSA and 0.2 ng/ml NGF, in the absence or presence of roscovitine (0.2, 2.0 or 10 μM). After 48 h, cells were fixed in 4% paraformaldehyde and immunostained with β-III tubulin antibody. Scale bar = 50 μM. **(B)** Axon lengths of DRG neurons exposed to different concentrations of roscovitine. Values are means ± SD of three independent experiments. Statistical significance using student’s *t*-test was set at *p* < 0.05*.

Repair of peripheral nerve injury normally requires axonal regrowth (Christie et al., 2014). Therefore, we examined the possibility that roscovitine can enhance axon regrowth in *ex vivo* adult primary neurons. For this experiment, adult male Sprague-Dawley rats were subjected to sciatic nerve transection at the mid-thigh region. After 3 days, L4 to L6 DRG were isolated from these rats, dissociated, and cultured to examine whether roscovitine will affect neurite outgrowth of these pre-injured primary sensory neurons. As with differentiating PC12 cells, we noted increased number and significantly (*p* < 0.05) longer neurites in cells treated with 0.2 μM roscovitine (Figures 2A,B). At this concentration, the average length of neurites was about twice those of neurons not treated with roscovitine. Interestingly, unlike in PC12 cells, increasing the concentration of roscovitine to 2 and 10 μM did not cause a pattern of increasing neurite length. DRG neuronal cell viability, upon addition of 2 and 10 μM roscovitine, trended toward a decline (data not shown). Although PC12 cells and primary neurons exhibit different sensitivities to roscovitine at 0.2–10 μM, it is clear that roscovitine can cause increased neurite length/axon regrowth in differentiating PC12 cells and preconditioned/pre-injured neurons.

### Roscovitine Increases the Number of Myelinated Axons that Repopulate Regenerating Rat Sciatic Nerve

**Figure 3:**
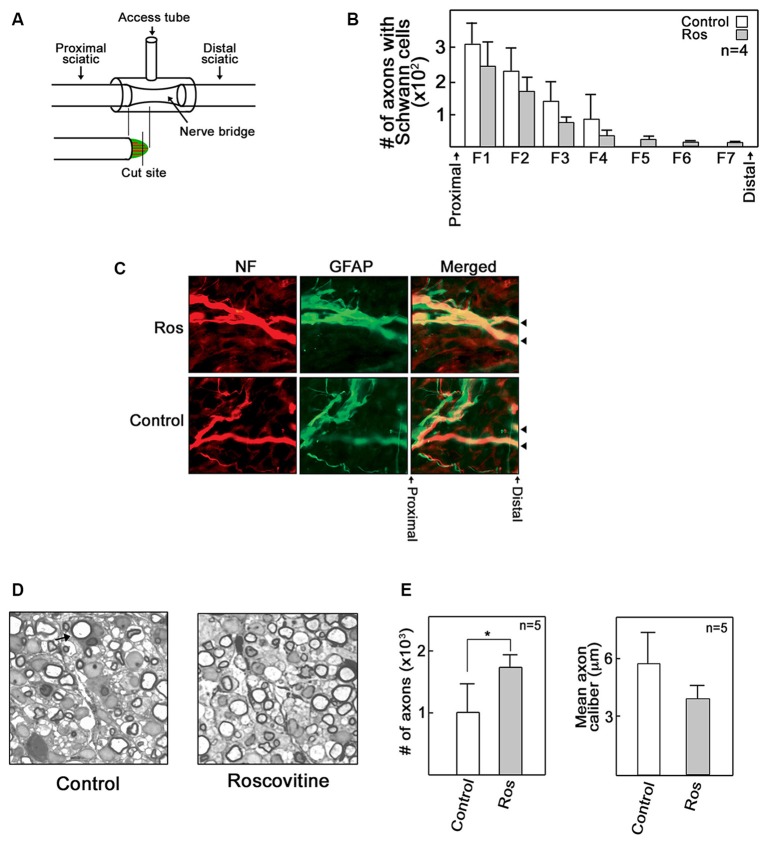
**Roscovitine increases the number of myelinated axons in regenerating rat sciatic nerve.** Adult male SD rats were subjected to sciatic nerve transection as described in “Materials and Methods” Section. **(A)** A schematic diagram showing the sciatic nerve lesion, the regeneration chamber and cutting site of EPON embedded section that lies perpendicular to the direction of the bridge. **(B)** Analysis of the number of axons with Schwann cells (SC) in control and roscovitine-treated rats 7 days after sciatic nerve transection. Axonal outgrowth were measured by serial counts (fields 1–7) proximal to the distal stump of a cut sciatic nerve by co-immunohistochemical staining for neurofilament (for neurons) and glial fibrillary acid protein (GFAP; for SC). Values are means ± SD; *n* = 4. Changes in values did not reach to statistical significance using Student’s *t*-test at *p* ≤ 0.05. **(C)** Representative image of axons with SC (examined in **B**) in control and roscovitine-treated rats at 7 days. Arrowheads are directed at axons. **(D)** Twenty-one days post-transection, axonal regeneration was evaluated by morphometric analysis of toluidine blue-stained EPON embedded cross sections of sciatic nerve bridges treated with Ringer’s solution (left panel) or 10 μM roscovitine (right panel). Arrow is directed at a myelinated axon. **(E)** Analysis of the number of myelinated axons (left panel) and axon caliber (right panel) in control (Ringer’s solution-treated) and roscovitine-treated rats 21 days after sciatic nerve transection. Values are means ± SD; *n* = 5. Statistical significance using student’s *t*-test was set at *p* < 0.05*.

Given our *in vitro* and *ex vivo* findings, we wondered whether roscovitine would improve axon regrowth in an adult rat *in vivo* regenerative model following total peripheral nerve trunk transection. A 3–5 mm gap was created after sciatic nerve transection and subsequent nerve retraction of the proximal and distal stumps; a scenario that simulates human nerve injury (Figure 3A). Administration of roscovitine was performed using a silicone conduit as described previously (McDonald and Zochodne, 2003). Repeated injections of 100 μl of 10 μM roscovitine or Ringer’s solution were performed on days 2, 4 and 6 (*n* = 9). Four of the nine rats were sacrificed for detection of early regenerating fibers on day 7 post-injury. The rest (*n* = 5) of the animals were analyzed for regenerating myelinated axons on day 21 post-injury. Since myelinated axons have not yet appeared on day 7 post-injury, we performed immunohistochemistry of longitudinal sections at this time point to obtain an index of early outgrowth when the earliest fibers begin to enter the regenerative stump. However, we found that roscovitine had no significant effect on early axon outgrowth in transected sciatic nerve 7 days after treatment (Figures 3B,C). On day 21 post-injury, immunohistochemistry imaging fields are crowded by a host of new fibers making discrete counting problematic. Thus, for day 21 post-injury samples, we analyzed EPON embedded semi-thin sections to appraise early and successful reconstitution of myelinated axons. Indeed, at 21 days following sciatic nerve transection, morphometric analysis of sections from the central portions of the nerve bridges revealed significantly (*p* < 0.05) increased numbers of repopulating myelinated axons in the roscovitine-treated group compared to the control group (Figures 3D,E left panel). We also assessed axon caliber but found no significant difference between the roscovitine-treated nerves and the control group. Overall, these observations extend our *in vitro* and *ex vivo* findings, indicating that roscovitine can be translated into a significant *in vivo* paradigm, with an impact on myelinated fiber repopulation of regenerating nerves.

### Roscovitine Interacts with Tubulin and Actin in Differentiating PC12 Cells

**Figure 4:**
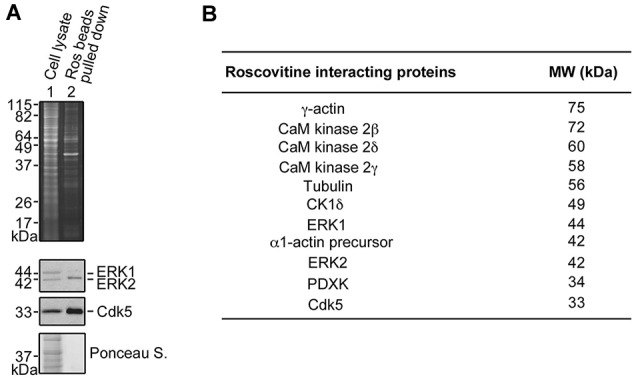
**Identification of roscovitine interacting proteins in differentiating PC12 cells. (A)** Neuronal differentiation of PC12 cells was induced using NGF. After pre-clearing with agarose beads, differentiating PC12 cell lysates were subjected to pull-down assay using roscovitine-agarose beads. The pulled-down proteins were analyzed by sodium dodecyl sulfate-polyacrylamide gel electrophoresis (SDS-PAGE) and stained with Sypro-Ruby Red (upper panel). A parallel SDSPAGE loaded with the same samples and analyzed by western blotting showed immunoreactivity to extracellular signal-regulated kinases 1/2 (ERK1/2) and Cdk5. **(B)** Pulled-down proteins were also analyzed by mass spectrometry. A list of roscovitine interacting proteins in differentiating PC12 cells treated with NGF and roscovitine is shown.

To understand the underlying molecular mechanism(s) behind roscovitine-induced increase in neurite outgrowth, axonal regrowth and peripheral nerve regeneration, we sought to identify the neuronal targets of roscovitine. To do so, lysates of differentiating PC12 cells treated with NGF and roscovitine were used for R-roscovitine-agarose pull-down assays. As shown in Figure 4A, after pre-clearing the cell lysates with agarose beads and mass spectrometry of the pulled down sample, we determined that the R-roscovitine-agarose beads effectively bound the known roscovitine targets, Cdk5 and ERK1/2 (Bach et al., 2005). We also detected binding of nine other known roscovitine targets, including calmodulin (CaM)-dependent kinases, casein kinase 1 (CK1) and pyridoxal kinase (PDXK; Bach et al., 2005; Figure 4B). However, we also detected two additional roscovitine-interacting proteins, tubulin and actin. Since peripheral nerve regeneration is dependent upon rapid polymerization of microtubules and dynamics of actin filaments in the growth cone (Kalil and Dent, 2005), we proceeded to characterize the interaction of roscovitine with tubulin and actin.

### Roscovitine Enhances Tubulin Polymerization

**Figure 5:**
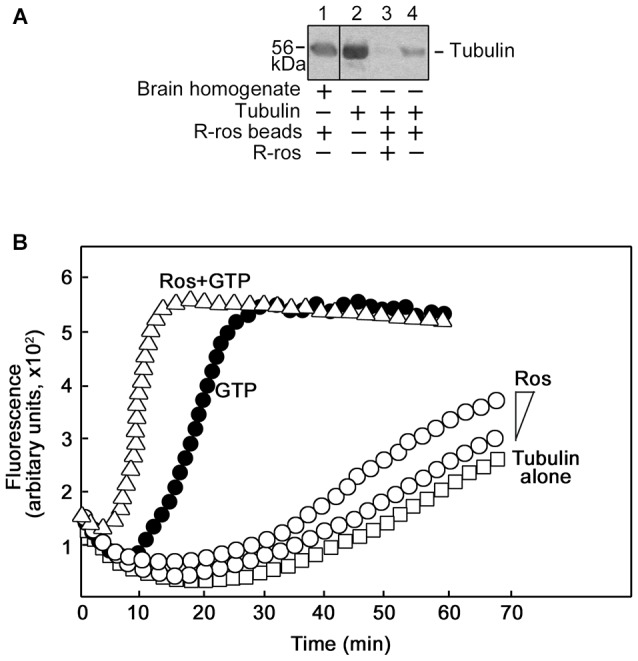
**Roscovitine interacts with tubulin directly, and enhances tubulin polymerization.** **(A)** Mouse brain homogenate (lane 1) and purified tubulin (lanes 3 and 4) were pre-cleared/-incubated with agarose beads and subsequently incubated with roscovitine-agarose beads. Tubulin binding to roscovitine beads was analyzed by western blotting using a tubulin antibody. Purified tubulin served as positive control (lane 2). For negative control (lane 3), tubulin was pre-incubated with 0.2 mM roscovitine (ros) prior to incubation with roscovitine-agarose beads. Note that no agarose-tubulin binding was observed after a second pre-clearing of brain homogenates. **(B)** Spectrophotometric tubulin polymerization assay was performed using a kit, and with slight modification of the manufacturer’s (Cytokeleton Inc.) protocol. To obtain detectable levels of polymerization, the recommendation for the *in vitro* tubulin polymerization assay kit is to use tubulin at 2 mg/ml in fluorescence buffer (80 mM PIPES pH 6.9, 2.0 mM MgCl_2_, 0.5 mM EGTA, 10 μM fluorescent reporter) + 1 mM Guanosine-5′-triphosphate (GTP). As R-roscovitine is a synthetic GTP analog, a comparable amount of R-roscovitine was used: 0.2 mM or 0.4 mM when used alone or 0.4 mM when used together with 0.5 mM GTP. Analysis of: polymerization of 4′,6-diamidino-2-phenylindole (DAPI)-tubulin alone (□) or (1) incubated with increasing concentrations of roscovitine (°; 0.2 mM or 0.4 mM); or (2) GTP (•, positive control; 0.5 mM), or GTP and roscovitine (Δ; 0.5 mM and 0.4 mM, respectively). Data from the two independent analyses with similar range of fluorescence values are shown together.

To characterize the interaction between tubulin and roscovitine, we initially performed R-roscovitine-agarose bead pull down assay of purified tubulin and mouse brain homogenates pre-incubated/-cleared with agarose beads. By western blotting (Figure 5A), we found that mouse brain homogenate tubulin (lane 1) and purified tubulin (lane 4) bound to the roscovitine beads while preincubation of purified tubulin with roscovitine prevents their interaction (lane 3), indicating that tubulin directly binds to roscovitine. We then examined whether roscovitine affects tubulin polymerization. To assess this possibility, we examined polymerization of tubulin in the presence and absence of roscovitine. As shown in Figure 5B, roscovitine by itself caused a dose-dependent increase in tubulin polymerization (°). Following normalization (subtraction of values at time 0), we detected more than 2-fold increase in tubulin polymerization upon addition of roscovitine alone. When added together with GTP, roscovitine caused earlier nucleation and enhanced rate (Δ) of GTP-dependent (•) tubulin polymerization. Indeed, the time needed to complete GTP-dependent polymerization was cut in half upon addition of roscovitine (15 min vs. 30 min).

### Roscovitine Enhances Arp2/3/Wave1-Mediated Actin Polymerization

**Figure 6:**
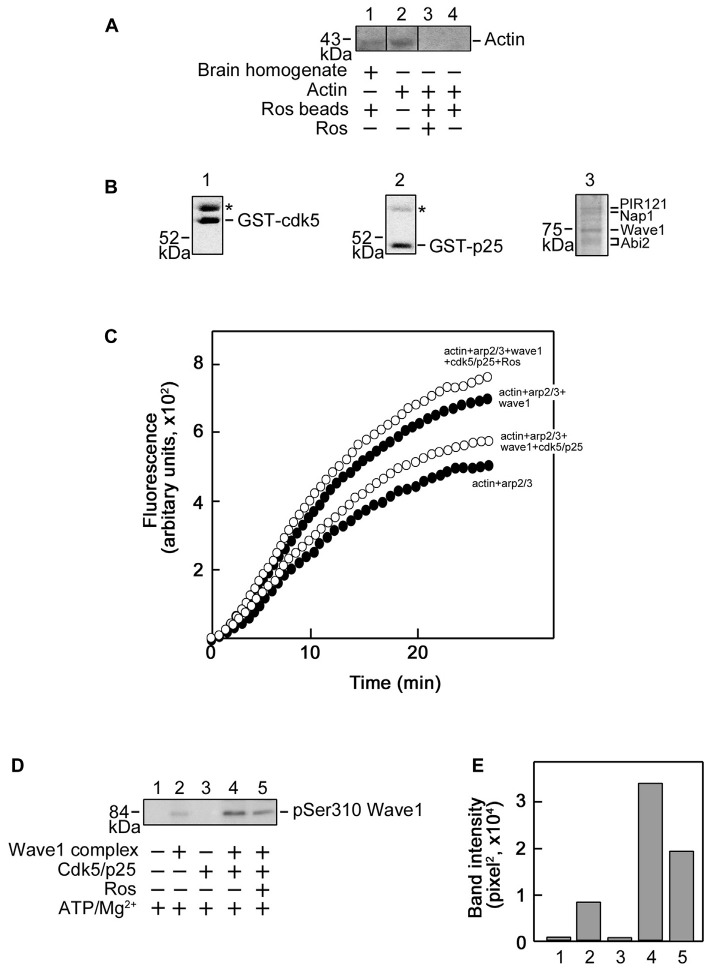
**Roscovitine interacts with actin indirectly, and enhances Actin-Related Proteins 2/3 (Arp2/3)/Wave1-mediated actin polymerization. (A)** Mouse brain homogenate (lane 1) and purified actin (lanes 3 and 4) were pre-cleared/-incubated with agarose beads and subsequently incubated with roscovitine-agarose beads. Actin binding to roscovitine beads was analyzed by western blotting using an actin antibody. Purified actin served as positive control (lane 2). For negative control (lane 3), actin was pre-incubated with 0.2 mM roscovitine (ros) prior to incubation with roscovitine-agarose beads. The lack of actin immunoreactivity in lane 4 indicates that actin in the brain homogenate (lane 1) interacts with roscovitine indirectly. Note that no agarose-actin binding was observed after a second pre-clearing of brain homogenates. **(B)** SDS-PAGE and Coomassie brilliant blue staining of purified Glutathione S-transferase-cyclin-dependent kinases 5 (GST-Cdk5; left panel), GST-p25 (middle panel; Cdk5 activator) and Strep-Wave1 complex (right panel). Asterisks correspond to the heat shock chaperon protein that copurifies with GST-Cdk5 and GST-p25. **(C)** Polymerization curve of pyrene-actin incubated with Arp2/3 ± Wave1 complex ± Cdk5/p25 ± preincubation with roscovitine. Values shown are obtained following a preincubation time of 11 min and fluorescence was set to an arbitrary value of zero. For all treatments, fluorescence start to plateau at ~27 min. **(D)** Western blot for Wave1 phospho-Ser310 showing that Cdk5/p25 phosphorylates Wave1 at Ser310 and this phosphorylation is inhibited by roscovitine. **(E)** Densitometric analysis of the representative blot in **(D)** using the NIH Image-J 1.61 software.

Next, we examined actin interaction with roscovitine with a further pull-down assay and western blot analysis. As shown in Figure 6A, although actin in pre-cleared mouse brain homogenates (lane 1) bound to the roscovitine beads, purified actin (lane 4) did not, indicating that actin (in brain homogenates) binds to roscovitine indirectly. Indeed, actin is known to bind to Cdk5 (Xu et al., 2011), a recognized direct target of roscovitine. Since Cdk5 has been shown to inhibit Arp2/3-dependent actin polymerization through phosphorylation of Wave1 (Kim et al., 2006), we examined whether roscovitine, a known Cdk5 inhibitor, could rescue actin polymerization. For this experiment, we used recombinant Cdk5 (Figure 6B, left panel), p25 (Cdk5 activator; Figure 6B, middle panel) and Wave1 (Figure 6B, right panel) that, as described previously (Kim et al., 2006), forms a complex and copurifies with endogenous PIR121, Nap1 and Abi2. We found that roscovitine, indeed, completely reversed the Cdk5/p25 inhibition of Arp2/3-Wave1-mediated actin polymerization (Figure 6C). Since under our experimental conditions, Cdk5/p25 clearly phosphorylates Wave1 (Figures 6D,E, lane 4 and bar 4, respectively), and roscovitine noticeably inhibits such phosphorylation (Figures 6D,E, lane 5 and bar 5, respectively), our results indicate that roscovitine-induced actin polymerization occurred through roscovitine interaction with Cdk5, which prevented Cdk5-mediated phosphorylation of Wave1 and subsequently, prevented Cdk5 inhibition of Arp2/3-Wave1-mediated actin polymerization.

### Roscovitine Causes Enhanced Activation of ERK1/2 and p38 MAPK in NGF-Treated Differentiating PC12 Cells

**Figure 7:**
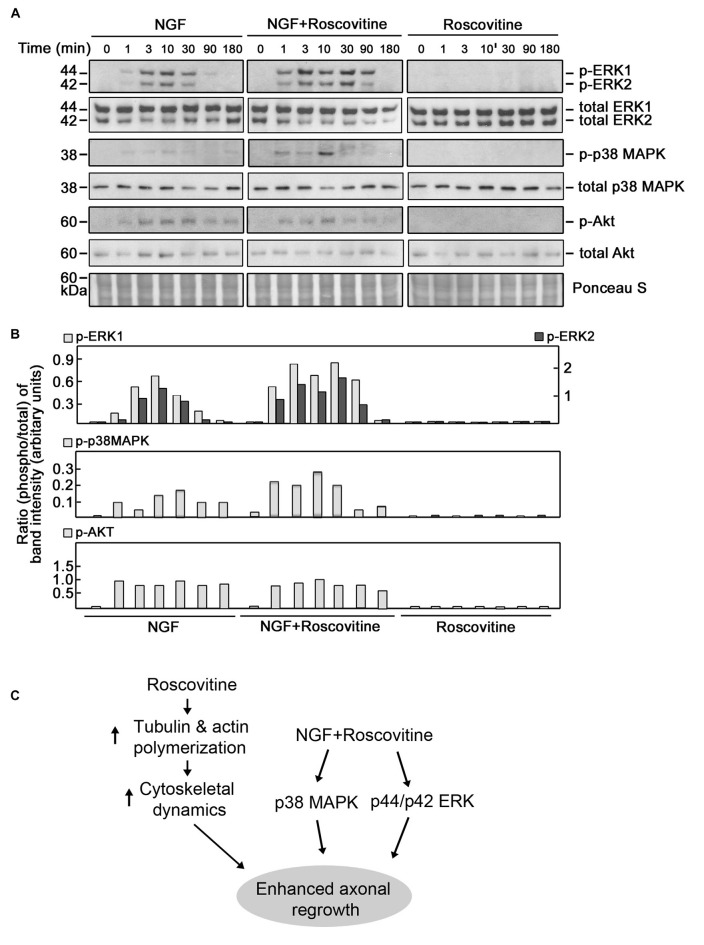
**Roscovitine enhances the activation of ERK1/2 and p38 mitogen-activated protein kinase (MAPK) in differentiating NGF-treated PC12 cells.** **(A)** Representative set of blots (from three sets/independent experiments) of lysates of PC12 cells plated and treated as described in “Materials and Methods” Section. After serum starvation for 18 h, cells were treated with NGF alone (10 ng/ml; left panel), roscovitine alone (10 μM; right panel) or NGF+roscovitine simultaneously (middle panel), and lysed at 0, 1, 3, 10, 30, 90 and 180 min post-treatment. Equal amounts of protein samples were then resolved in 12.5% SDS-PAGE and subjected to western blot analysis using phospho-antibodies against ERK1/2, p38 MAPK and AKT. Additional test for loading equivalent amounts of protein was performed by Ponceau S staining. **(B)** The ratios of the band intensities [from the representative blots in **(A)**] of phospho-ERK1/2, phospho-p38 MAPK and phospho-Akt vs. total ERK, total p38 MAPK and total Akt, respectively, were determined following densitometric scanning of bands using the NIH Image-J 1.61 software. Note the different Y-axis scales for p-ERK1/total ERK (left) and p-ERK2/total ERK (right). Analysis of the ratios of the band intensities of phospho-AKT vs. total AKT did not show any considerable changes at different time points in the three treatment groups. **(C)** Proposed molecular mechanisms by which roscovitine enhances NGF-induced axonal regrowth. Roscovitine promotes axonal regrowth by inducing tubulin and actin polymerization (left panel). In the presence of NGF, roscovitine enhances axonal regrowth through activation of the ERK1/2 and p38 MAPK pathways.

ERK1/2, p38 MAPK and AKT have been implicated in neurite outgrowth and axonal regeneration (Agthong et al., 2009; Okada et al., 2011; Wang et al., 2011; Kato et al., 2013; Sarina et al., 2013; Mufti et al., 2014). Therefore, we wondered whether increased axon regrowth resulting from roscovitine treatment is due to synergistic activation of ERK1/2, p38 MAPK and/or AKT signaling pathways in addition to enhancement of tubulin and actin polymerization. For this experiment, we again used PC12 cells as a substantial number of injured rats would be required to obtain a sufficient number of DRG neurons for several western blots. Lysates of differentiating PC12 cells grown in the presence of NGF with or without roscovitine were examined for the phosphorylation levels of ERK1/2, p38 MAPK and AKT. As shown in Figures 7A,B, simultaneous treatment with NGF and roscovitine (middle panels) caused considerable activation of ERK1/2 and p38 MAPK at an earlier time point (1 min) compared to cells treated with NGF alone (3 min; left panels). In addition, we observed enhanced and prolonged activation of ERK1/2 in cells treated with NGF + roscovitine (90 min) compared to cells treated with roscovitine alone (30 min). While we also noted AKT activation following treatment with NGF, addition of roscovitine did not cause a change in activation of this kinase.

## Discussion

It is recognized that triggering the cell cycle plays a role in post-mitotic cell death. Interestingly, activation of the cell cycle has also been implicated in secondary damage following injury to the spinal cord (Byrnes et al., 2007). Consequently, blocking major cell cycle signaling molecules inhibits not only cell proliferation but also injury-related cell death. For example, in rodent spinal cord injury models, it was shown that inhibiting the cell cycle results in a reduction of both neuronal death and inflammation, and subsequently, improved sensory motor recovery (Wu et al., 2011). The synthetic purine analog, roscovitine, has been shown to regulate both the cell cycle and inflammatory process but a potential role in sensory motor neuron recovery has not yet been investigated.

Our current findings suggest that roscovitine, which inhibits cell cycle Cdks, has the ability to promote nerve regeneration. In this study, myelinated axons were chosen as an index of maturating fibers and to establish a correlation with functional outcome. While early measurements in a nerve conduit and *in vitro* are unmyelinated as we assessed by day 7, some are destined to grow in caliber and become myelinated. Thus, this population of fibers (early unmyelinated) does not necessarily predict later myelinated axon repopulation of a mature nerve. Unmyelinated counts can also be complex later since they are a mix of fibers destined to remain unmyelinated and axons that will be myelinated later. By focusing on myelinated axons, we believe we have a better index of the population that translates into functional recovery. As shown in our adult rat *in vivo* regenerative model system, treatment with low-dose roscovitine following total peripheral nerve trunk transection caused an increase in number of repopulating myelinated axons. Although we could not disregard the fact that roscovitine binds to several ATP-dependent protein targets, and principally, GTP- or NAD-dependent protein kinases (Bach et al., 2005), its ability to interact with tubulin and actin, may be a major mechanism in its ability to promote axonal regeneration. It is possible that inosine effect on collateral axon growth (Dachir et al., 2014) and adenosine and/or guanosine effects on axonal degeneration and axon preservation after injury (Press and Milbrandt, 2009) occur through a similar mechanism.

Indeed, we found that roscovitine binds to tubulin directly and such interaction causes tubulin polymerization that is independent of GTP. In the presence of GTP, roscovitine has the ability to induce earlier nucleation and enhance the rate of tubulin polymerization. This may be explained by roscovitine binding to the tubulin exchangeable nucleotide-binding site (E-site) with high affinity (De Azevedo et al., 1997; Gray et al., 1999). Consistent with this suggestion, it has been shown that an analog of GTP, guanylyl-(alpha, beta)-methylene-diphosphonate (GMPCPP), which is insensitive to hydrolyzation, favors polymerization by strongly promoting spontaneous nucleation of microtubules (Hyman et al., 1992) which is critical for the early stages of microtubule assembly (Wang et al., 2005).

On the other hand, roscovitine does not bind actin directly. Therefore, its ability to enhance actin polymerization is likely due to its interaction with other intermediate proteins. Indeed, Cdk5, a direct target of roscovitine (Bach et al., 2005), has the ability to bind actin (Xu et al., 2011). Previously, Kim et al. (2006) reported that WAVE1 phosphorylation by Cdk5 directly interferes with Arp2/3-dependent actin polymerization, which is known to be critical for growth cone formation. Since roscovitine is a potent inhibitor of Cdk5, roscovitine binding and inhibition of Cdk5 activity could account for the enhanced polymerization effect on actin by roscovitine. Incidentally, this further supports the notion that Cdk5 serves as an inhibitor of actin polymerization (Kim et al., 2006). Thus, our findings suggest that roscovitine promotes not only tubulin-dependent neuronal functions but also actin-dependent functions. These roscovitine roles may, indeed, occur during roscovitine-induced neurite outgrowth, *ex vivo* axon regrowth and regeneration of injured sciatic nerve in rats.

It is interesting that, although PC12 cells exhibit a dose-dependent increase in neurite outgrowth from 0.2 to 5 μM roscovitine, primary DRG sensory neurons isolated following sciatic nerve transection showed a more enhanced axon regrowth at a lower roscovitine concentration. In other words, axonal regeneration in pre-injured primary DRG sensory neurons is greater at 0.2 μM than at 2 μM roscovitine, and 2 μM elicited greater axon regrowth than 10 μM. This appears to suggest that primary DRG sensory neurons have greater uptake ability for roscovitine compared to differentiating PC12 cells, or that the primary DRG sensory neurons have better representation of neural properties. It is also possible that increased sensitivity of DRG neurons to roscovitine is due to the difference in the required culture conditions to induce neurite outgrowth. While PC12 cells were cultured in the presence of 10 ng/ml NGF to induce differentiation, DRG primary neurons are maintained in media containing 0.2 ng/ml NGF. Thus, it appears that with the higher amount of NGF in PC12 cell cultures, which can already cause neurite outgrowth, a higher concentration of roscovitine is needed to see an enhanced effect on neurite outgrowth. Conversely, a relatively lower concentration of roscovitine is needed in DRG neuronal cultures, which are grown in low NGF media, to see an axon regrowth promoting effect. Nonetheless, our findings indicate that roscovitine has a parallel impact on neurite/axon regrowth in PC12 cells and in preconditioned/pre-injured neurons.

ERK1/2, p38 MAPK and AKT have all been implicated in neurite outgrowth and axonal regeneration (Agthong et al., 2009; Okada et al., 2011; Wang et al., 2011; Kato et al., 2013; Sarina et al., 2013). In this study, we also found that these kinases are activated in differentiating PC12 cells. However, we further found that roscovitine causes earlier activation of ERK1/2 and p38 MAPK as well as prolonged activation of ERK1/2. This is not surprising as roscovitine’s IC50 values for ERK1, ERK2 and p38 MAPK are quite high as indicated above. Thus, while roscovitine may inhibit MAPKs at high concentrations (i.e., greater than their respective IC50 values) the low-dose roscovitine that we used (10 μM) has no inhibitory effect but instead, enhanced the activation of ERK1/2 and p38 MAPK. These findings suggest that both ERK1/2 and p38 MAPK are involved in roscovitine-induced neurite outgrowth and axonal regeneration. Indeed, there has been an earlier report that activation of ERK1/2 and p38 MAPK is linked to neurite outgrowth in PC12 cells (Sarina et al., 2013). Thus, consistent with our findings, we propose a model (Figure 7C) whereby roscovitine promotes axonal regrowth through induction of tubulin and actin polymerization. In the presence of neurotrophic factors, such as NGF, axonal regrowth is enhanced by roscovitine via increased activation of the p38 MAPK and ERK1/2 signaling pathways.

Interestingly, the ERK1/2 pathway has been associated with the promotion of dedifferentiation of myelinating SC (Jessen and Mirsky, 2008; Napoli et al., 2012), a process that supports axon regeneration. In addition, p38 MAPK activation induces denervated SC phenotype and negatively regulates SC differentiation and myelination (Yang et al., 2012). However, once axons have started to regenerate, SC redifferentiate and remyelinate. Thus, our finding that enhanced activation of ERK1/2 and p38 MAPK play important roles in neurite outgrowth in neuronal cultures complements previous suggestions that these kinases are key regulators of axonal regeneration.

Certainly, roscovitine-induced actin and tubulin polymerization may explain our *in vitro*, *ex vivo and in vivo* data, demonstrating enhancement of neurite outgrowth in neuronal-like PC12 cells, axonal regrowth in primary DRG sensory neurons and induction of regeneration of transected rat sciatic nerve, respectively. While it should be noted that depolymerization is at least as important as polymerization in mobilizing microtubular plasticity in the growth cone, our premise that the ability of roscovitine to enhance tubulin polymerization is linked to its ability to induce regeneration of injured rat sciatic nerve is consistent with recent reports. Indeed, the anti-cancer agents, paclitaxel (Hellal et al., 2011) and epothilone B (Ruschel et al., 2015), both of which stabilize microtubules, promote axon regrowth. Furthermore, the ability of epothilone B to induce axon regrowth was shown to cause restoration of locomotive function in a rat spinal cord injury model (Ruschel et al., 2015). Interestingly, roscovitine is currently under phase II clinical trial in combination with standard chemotherapy regimens for advanced breast cancer and stage IIIB/IV non-small cell lung cancer (Aldoss et al., 2009; Nair et al., 2011). It appears that the microtubule-mediated mechanisms of roscovitine- and epothilone B-induced nerve repair are similar.

Taken together, our findings point to a therapeutic potential of roscovitine for peripheral nerve injury. Therefore, the next step is to assess this potential by initial functional recovery tests in animals and, if promising, subsequently in human clinical testing. If, indeed, promising, much remains to be explored on the potential use of roscovitine as therapy for peripheral nerve injury, aside from appropriate route of administration, toxicity profile and maximum tolerated dose. Given its role in axon protection and regrowth, and potential for cancer therapy, roscovitine may simultaneously offer some protection against chemotherapy-induced neuropathy, a possibility that is worth exploring.

## Author Contributions

VL performed most of the experiments and wrote a draft of the manuscript. SD performed the experiments in Figure 2. JLR, DZ and K-YL conceived the idea for the project and contributed to writing the final manuscript. M-YJ contributed to the design of an experiment and discussion about the project.

## Funding

This work was supported in part by grants from the Canadian Institutes of Health Research (MOP-123400), Natural Sciences and Engineering Research Council of Canada (NSERC) (RGPIN/312985-2011) and the Korean Ministry of Education, Science and Technology to K-YL; NSERC (RGPIN/356448-2008) to JLR; the WCU program (R31-20004) through the NRFK funded by the Korean Ministry of Education, Science and Technology to M-YJ.

## Conflict of Interest Statement

The authors declare that the research was conducted in the absence of any commercial or financial relationships that could be construed as a potential conflict of interest.

## References

[B1] Agthong S. Koonam J. Kaewsema A. Chentanez V. (2009). Inhibition of MAPK ERK impairs axonal regeneration without an effect on neuronal loss after nerve injury. Neurol. Res. 31, 1068–1074. 10.1179/174313209X380883 19426585

[B2] Aldoss I. T. Tashi T. Ganti A. K. (2009). Seliciclib in malignancies. Expert Opin. Investig. Drugs 18, 1957–1965. 10.1517/13543780903418445 19938906

[B3] Bach S. Knockaert M. Reinhardt J. Lozach O. Schmitt S. Baratte B.. (2005). Roscovitine targets, protein kinases and pyridoxal kinase. J. Biol. Chem. 280, 31208–31219. 10.1074/jbc.M500806200 15975926

[B4] Byrnes K. R. Stoica B. A. Fricke S. Di Giovanni S. Faden A. I. (2007). Cell cycle activation contributes to post-mitotic cell death and secondary damage after spinal cord injury. Brain 130, 2977–2992. 10.1093/brain/awm179 17690131

[B5] Carter J. M. Demizieux L. Campenot R. B. Vance D. E. Vance J. E. (2008). Phosphatidylcholine biosynthesis via CTP: phosphocholine cytidylyltransferase 2 facilitates neurite outgrowth and branching. J. Biol. Chem. 283, 202–212. 10.1074/jbc.M706531200 17981805

[B6] Chierzi S. Ratto G. M. Verma P. Fawcett J. W. (2005). The ability of axons to regenerate their growth cones depends on axonal type and age and is regulated by calcium, cAMP and ERK. Eur. J. Neurosci. 21, 2051–2062. 10.1111/j.1460-9568.2005.04066.x 15869501

[B7] Christie K. J. Krishnan A. Martinez J. A. Purdy K. Singh B. Eaton S.. (2014). Enhancing adult nerve regeneration through the knockdown of retinoblastoma protein. Nat. Commun. 5:3670. 10.1038/ncomms4670 24752312 PMC5028199

[B8] Christie K. J. Webber C. A. Martinez J. A. Singh B. Zochodne D. W. (2010). PTEN inhibition to facilitate intrinsic regenerative outgrowth of adult peripheral axons. J. Neurosci. 30, 9306–9315. 10.1523/JNEUROSCI.6271-09.2010 20610765 PMC6632469

[B9] Dachir S. Shabashov D. Trembovler V. Alexandrovich A. G. Benowitz L. I. Shohami E. (2014). Inosine improves functional recovery after experimental traumatic brain injury. Brain Res. 1555, 78–88. 10.1016/j.brainres.2014.01.044 24502983

[B10] De Azevedo W. F. Leclerc S. Meijer L. Havlicek L. Strnad M. Kim S. H. (1997). Inhibition of cyclin-dependent kinases by purine analogues: crystal structure of human cdk2 complexed with roscovitine. Eur. J. Biochem. 243, 518–526. 10.1111/j.1432-1033.1997.0518a.x 9030780

[B11] De S. Trigueros M. A. Kalyvas A. David S. (2003). Phospholipase A2 plays an important role in myelin breakdown and phagocytosis during wallerian degeneration. Mol. Cell. Neurosci. 24, 753–765. 10.1016/s1044-7431(03)00241-0 14664823

[B12] Eng H. Lund K. Campenot R. B. (1999). Synthesis of β-tubulin, actin and other proteins in axons of sympathetic neurons in compartmented cultures. J. Neurosci. 19, 1–9. 9870932 10.1523/JNEUROSCI.19-01-00001.1999 PMC6782370

[B13] Gao J. X. Zhou Y. Q. Zhang R. H. Ma X. L. Liu K. J. (2005). Caspase-3 plays a required role in PC12 cell apoptotic death induced by roscovitine. Sheng Li Xue Bao 57, 755–760. 16344902

[B14] Gray N. Detivaud L. Doerig C. Meijer L. (1999). ATP-site directed inhibitors of cyclin-dependent kinases. Curr. Med. Chem. 6, 859–875. 10495356

[B15] Hamilton G. Klameth L. Rath B. Thalhammer T. (2014). Synergism of cyclin-dependent kinase inhibitors with camptothecin derivatives in small cell lung cancer cell lines. Molecules 19, 2077–2088. 10.3390/molecules19022077 24549232 PMC6271949

[B16] Harada T. Morooka T. Ogawa S. Nishida E. (2001). ERK induces p35, a neuron-specific activator of Cdk5, through induction of Egr1. Nat. Cell Biol. 3, 453–459. 10.1038/35074516 11331872

[B17] Hellal F. Hurtado A. Ruschel J. Flynn K. C. Laskowski C. J. Umlauf M.. (2011). Microtubule stabilization reduces scarring and causes axon regeneration after spinal cord injury. Science 331, 928–931. 10.1126/science.1201148 21273450 PMC3330754

[B18] Hui A. B. Yue S. Shi W. Alajez N. M. Ito E. Green S. R.. (2009). Therapeutic efficacy of seliciclib in combination with ionizing radiation for human nasopharyngeal carcinoma. Clin. Cancer Res. 15, 3716–3724. 10.1158/1078-0432.CCR-08-2790 19470731

[B19] Hyman A. A. Salser S. Drechsel D. N. Unwin N. Mitchison T. J. (1992). Role of GTP hydrolysis in microtubule dynamics: information from a slowly hydrolyzable analogue, GMPCPP. Mol. Biol. Cell 3, 1155–1167. 10.1091/mbc.3.10.1155 1421572 PMC275679

[B20] Jessen K. R. Mirsky R. (2008). Negative regulation of myelination: relevance for development, injury and demyelinating disease. Glia 56, 1552–1565. 10.1002/glia.20761 18803323

[B21] Kalil K. Dent E. W. (2005). Touch and go: guidance cues signal to the growth cone cytoskeleton. Curr. Opin. Neurobiol. 15, 521–526. 10.1016/j.conb.2005.08.005 16143510

[B22] Kato N. Matsumoto M. Kogawa M. Atkins G. J. Findlay D. M. Fujikawa T.. (2013). Critical role of p38 MAPK for regeneration of the sciatic nerve following crush injury *in vivo*. J. Neuroinflammation 10:1. 10.1186/1742-2094-10-1 23282009 PMC3541116

[B23] Kim Y. Sung J. Y. Ceglia I. Lee K. W. Ahn J. H. Halford J. M.. (2006). Phosphorylation of WAVE1 regulates actin polymerization and dendritic spine morphology. Nature 442, 814–817. 10.1038/nature04976 16862120

[B24] Latreille M. Abu-Thuraia A. Oliva R. Zuo D. Larose L. (2012). Casein kinase igamma2 impairs fibroblasts actin stress fibers formation and delays cell cycle progression in g1. Int. J. Cell Biol. 2012:684684. 10.1155/2012/684684 22496693 PMC3312262

[B25] Lee J. H. Kim K. T. (2004). Induction of cyclin-dependent kinase 5 and its activator p35 through the extracellular-signal-regulated kinase and protein kinase A pathways during retinoic-acid mediated neuronal differentiation in human neuroblastoma SK-N-BE(2)C cells. J. Neurochem. 91, 634–647. 10.1111/j.1471-4159.2004.02770.x 15485494

[B26] Liu N. A. Araki T. Cuevas-Ramos D. Hong J. Ben-Shlomo A. Tone Y.. (2015). Cyclin E-mediated human proopiomelanocortin regulation as a therapeutic target for cushing disease. J. Clin. Endocrinol. Metab. 100, 2557–2564. 10.1210/jc.2015-1606 25942479 PMC5393529

[B27] McDonald D. S. Zochodne D. W. (2003). An injectable nerve regeneration chamber for studies of unstable soluble growth factors. J. Neurosci. Methods 122, 171–178. 10.1016/s0165-0270(02)00319-9 12573476

[B28] Meijer L. Borgne A. Mulner O. Chong J. P. Blow J. J. Inagaki N.. (1997). Biochemical and cellular effects of roscovitine, a potent and selective inhibitor of the cyclin-dependent kinases cdc2, cdk2 and cdk5. Eur. J. Biochem. 243, 527–536. 10.1111/j.1432-1033.1997.t01-2-00527.x 9030781

[B29] Moutinho-Pereira S. Matos I. Maiato H. (2010). *Drosophila* S2 cells as a model system to investigate mitotic spindle dynamics, architecture and function. Methods Cell Biol. 97, 243–257. 10.1016/S0091-679X(10)97014-3 20719275

[B30] Mufti R. E. Sarker K. Jin Y. Fu S. Rosales J. L. Lee K. Y. (2014). Thrombin enhances NGF-mediated neurite extension via increased and sustained activation of p44/42 MAPK and p38 MAPK. PLoS One 9:e103530. 10.1371/journal.pone.0103530 25061982 PMC4111596

[B31] Nair B. C. Vallabhaneni S. Tekmal R. R. Vadlamudi R. K. (2011). Roscovitine confers tumor suppressive effect on therapy-resistant breast tumor cells. Breast Cancer Res. 13:R80. 10.1186/bcr2929 21834972 PMC3218960

[B32] Napoli I. Noon L. A. Ribeiro S. Kerai A. P. Parrinello S. Rosenberg L. H.. (2012). A central role for the ERK-signaling pathway in controlling Schwann cell plasticity and peripheral nerve regeneration *in vivo*. Neuron 73, 729–742. 10.1016/j.neuron.2011.11.031 22365547

[B33] Nutley B. P. Raynaud F. I. Wilson S. C. Fischer P. M. Hayes A. Goddard P. M.. (2005). Metabolism and pharmacokinetics of the cyclin-dependent kinase inhibitor R-roscovitine in the mouse. Mol. Cancer Ther. 4, 125–139. 15657360

[B34] Okada K. Tanaka H. Temporin K. Okamoto M. Kuroda Y. Moritomo H.. (2011). Akt/mammalian target of rapamycin signaling pathway regulates neurite outgrowth in cerebellar granule neurons stimulated by methylcobalamin. Neurosci. Lett. 495, 201–204. 10.1016/j.neulet.2011.03.065 21458538

[B35] Planchais S. Glab N. Tréhin C. Perennes C. Bureau J. M. Meijer L.. (1997). Roscovitine, a novel cyclin-dependent kinase inhibitor, characterizes restriction point and G2/M transition in tobacco BY-2 cell suspension. Plant J. 12, 191–202. 10.1046/j.1365-313x.1997.12010191.x 9263460

[B36] Press C. Milbrandt J. (2009). The purine nucleosides adenosine and guanosine delay axonal degeneration *in vitro*. J. Neurochem. 109, 595–602. 10.1111/j.1471-4159.2009.06002.x 19245660 PMC2682787

[B37] Raynaud F. I. Whittaker S. R. Fischer P. M. McClue S. Walton M. I. Barrie S. E.. (2005). *In vitro* and *in vivo* pharmacokinetic-pharmacodynamic relationships for the trisubstituted aminopurine cyclin-dependent kinase inhibitors olomoucine, bohemine and CYC202. Clin. Cancer Res. 11, 4875–4887. 10.1158/1078-0432.ccr-04-2264 16000586

[B38] Richardson P. M. Miao T. Wu D. Zhang Y. Yeh J. Bo X. (2009). Responses of the nerve cell body to axotomy. Neurosurgery 65, A74–A79. 10.1227/01.NEU.0000352378.26755.C3 19927082

[B39] Ruschel J. Hellal F. Flynn K. C. Dupraz S. Elliott D. A. Tedeschi A.. (2015). Axonal regeneration. Systemic administration of epothilone B promotes axon regeneration after spinal cord injury. Science 348, 347–352. 10.1126/science.aaa2958 25765066 PMC4445125

[B40] Sarina Yagi Y. Nakano O. Hashimoto T. Kimura K. Asakawa Y.. (2013). Induction of neurite outgrowth in PC12 cells by artemisinin through activation of ERK and p38 MAPK signaling pathways. Brain Res. 1490, 61–71. 10.1016/j.brainres.2012.10.059 23123209

[B41] Stoll G. Jander S. Myers R. R. (2002). Degeneration and regeneration of the peripheral nervous system: from Augustus Waller’s observations to neuroinflammation. J. Peripher. Nerv. Syst. 7, 13–27. 10.1046/j.1529-8027.2002.02002.x 11939348

[B42] van Engeland M. Kuijpers H. J. Ramaekers F. C. Reutelingsperger C. P. Schutte B. (1997). Plasma membrane alterations and cytoskeletal changes in apoptosis. Exp. Cell Res. 235, 421–430. 10.1006/excr.1997.3738 9299167

[B43] Vita M. Meurling L. Pettersson T. Cruz-Sidén M. Sidén A. Hassan M. (2004). Analysis of roscovitine using novel high performance liquid chromatography and UV-detection method: pharmacokinetics of roscovitine in rat. J. Pharm. Biomed. Anal. 34, 425–431. 10.1016/s0731-7085(03)00534-x 15013157

[B44] Vogelaar C. F. Gervasi N. M. Gumy L. F. Story D. J. Raha-Chowdhury R. Leung K. M.. (2009). Axonal mRNAs: characterisation and role in the growth and regeneration of dorsal root ganglion axons and growth cones. Mol. Cell. Neurosci. 42, 102–115. 10.1016/j.mcn.2009.06.002 19520167 PMC4603359

[B45] Wang H. W. Long S. Finley K. R. Nogales E. (2005). Assembly of GMPCPP-bound tubulin into helical ribbons and tubes and effect of colchicine. Cell Cycle 4, 1157–1160. 10.4161/cc.4.9.2042 16123589

[B46] Wang X. Wang Z. Yao Y. Li J. Zhang X. Li C.. (2011). Essential role of ERK activation in neurite outgrowth induced by alpha-lipoic acid. Biochim. Biophys. Acta 1813, 827–838. 10.1016/j.bbamcr.2011.01.027 21295083

[B47] Wu J. Stoica B. A. Faden A. I. (2011). Cell cycle activation and spinal cord injury. Neurotherapeutics 8, 221–228. 10.1007/s13311-011-0028-2 21373950 PMC3101833

[B48] Xu J. Tsutsumi K. Tokuraku K. Estes K. A. Hisanaga S. Ikezu T. (2011). Actin interaction and regulation of cyclin-dependent kinase 5/p35 complex activity. J. Neurochem. 116, 192–204. 10.1111/j.1471-4159.2010.06824.x 20492361 PMC2957558

[B49] Yamazaki T. Sabit H. Oya T. Ishii Y. Hamashima T. Tokunaga A.. (2009). Activation of MAP kinases, Akt and PDGF receptors in injured peripheral nerves. J. Peripher. Nerv. Syst. 14, 165–176. 10.1111/j.1529-8027.2009.00228.x 19909480

[B50] Yang D. P. Kim J. Syed N. Tung Y. J. Bhaskaran A. Mindos T.. (2012). p38 MAPK activation promotes denervated Schwann cell phenotype and functions as a negative regulator of Schwann cell differentiation and myelination. J. Neurosci. 32, 7158–7168. 10.1523/JNEUROSCI.5812-11.2012 22623660 PMC3369433

[B51] Zheng Y. L. Li B. S. Kanungo J. Kesavapany S. Amin N. Grant P.. (2007). Cdk5 Modulation of mitogen-activated protein kinase signaling regulates neuronal survival. Mol. Biol. Cell 18, 404–413. 10.1091/mbc.e06-09-0851 17108320 PMC1783783

